# Minimizing the “Kitchen Sink” Approach: De-implementation of Unnecessary Care for Patients with Anaphylaxis in a Pediatric Emergency Department

**DOI:** 10.1097/pq9.0000000000000535

**Published:** 2022-03-30

**Authors:** Laura Vanston, Kaleigh Ogawa, Julia Freeman, Maureen Bauer, Kirsten Carel, Irina Topoz

**Affiliations:** From the 1 Section of Pediatric Emergency Medicine, Children’s Hospital Colorado, University of Colorado School of Medicine, Aurora, Colo.; 2 Clinical Effectiveness, Children’s Hospital Colorado, Aurora, Colo.; 3 University of Colorado School of Medicine, Section of Allergy and Immunology, Children’s Hospital Colorado, Aurora, Colo..

## Abstract

Epinephrine is the only medication that prevents morbidity and mortality in anaphylaxis. Systemic corticosteroids and H2 receptor antagonists (H2RA) may benefit select cases but are not universally indicated. This study aims to de-implement the universal use of steroids and H2RAs and emphasize epinephrine-focused care for children with anaphylaxis during acute care visits. The study aims to reduce steroid and H2RA use from 81% and 60%, respectively, to 30% by December 2019.

**Methods:**

The primary outcome measures were the percent of patients receiving steroids and H2RAs in the emergency department (ED) or urgent care (UC). Process measure was the frequency of intravenous (IV) line placement. Balancing measures were ED/UC length of stay, admission rate, and ED/UC return visit rate. In addition, a multidisciplinary team designed the following interventions: (1) anaphylaxis clinical pathway to emphasize epinephrine-focused care, outline criteria for second-line therapies and a provider guideline for ED/UC observation; (2) standardize unit-based anaphylaxis medication kits; (3) optimize electronic medical record tools, including order sets and discharge instructions to be concordant with guideline recommendations.

**Results:**

The study included 870 patients. There was special cause variation in the use of steroids (81%–33%) and H2RAs (60%–11%), ED/UC Length of stay decreased (6.2–5.0 hours). There was no special cause variation in admission rates or ED/UC return visit rates.

**Conclusion:**

Universal use of systemic steroids and H2RAs can be safely de-implemented in pediatric patients with anaphylaxis using quality improvement methods.

## INTRODUCTION

Emergency department (ED) visits for anaphylaxis have been steadily increasing, particularly in the pediatric population.^1,2^ Diagnosis and treatment of anaphylaxis remain highly variable among healthcare providers.^3,4^ The American Academy of Asthma, Allergy, and Immunology (AAAAI) anaphylaxis national guidelines state that epinephrine is the only medication that prevents morbidity and mortality.^5,6^ Use of corticosteroids and antihistamines, including histamine-1 (H1) and histamine-2 (H2) receptor antagonists, is widespread, but there is insufficient evidence that these medications improve outcomes, including biphasic reactions.^7–9^

Before this study period, children seen within our acute care network with suspected anaphylaxis routinely received combination therapy, including epinephrine, systemic steroids, and H1 and H2 receptor antagonists. The primary goal of this study was to de-implement unnecessary use of adjunct medications and emphasize epinephrine-focused care for children with anaphylaxis. We aimed to reduce the use of steroid and H2 receptor antagonists (H2RA) from 81% and 60%, respectively, to 30% by December 2019.

## METHODS

### Population and Setting

This study took place at a single institution pediatric acute care network consisting of 6 urgent and emergency care locations in urban and suburban areas. The network included an ED within a freestanding pediatric tertiary care center and community emergency and urgent care (UC) locations. The combined annual patient volume was around 160,000. During this study, three sites were staffed with pediatric emergency medicine (PEM) physicians, general pediatricians, and advanced practice providers. In comparison, staffing at the other three sites included general pediatricians and advanced practice providers. In addition, four sites offered observation and inpatient services. All sites shared an electronic health record system.

This study included all patients from 3 months to 21 years of age who presented with suspected anaphylaxis to our acute care network. The study excluded patients under 3 months of age, patients with symptoms attributable to other causes, and patients receiving a blood transfusion during an ED visit. The study was approved by the Children’s Hospital Colorado Organizational Research Risk and QI Review Panel.

## IMPROVEMENT STRATEGY AND INTERVENTIONS

### Multidisciplinary Team

Project leads performed stakeholder analysis to identify quality improvement (QI) team members. As a result, the QI team included pediatric nurse practitioners, PEM physicians, allergy, and immunology physicians, a process improvement expert, pediatric nurses, general pediatricians, hospital medicine physicians, pharmacists, and information technology experts.

### Improvement Methods

We used Lean methodology for this study. Specifically, we used the SCORE (Select, Clarify, Organize, Run, Evaluate) framework to understand the current state, establish goals, and identify and implement interventions.^10^ The SCORE framework supports implementing multiple solutions at the same time rather than consecutively.

**Fig. 1.**
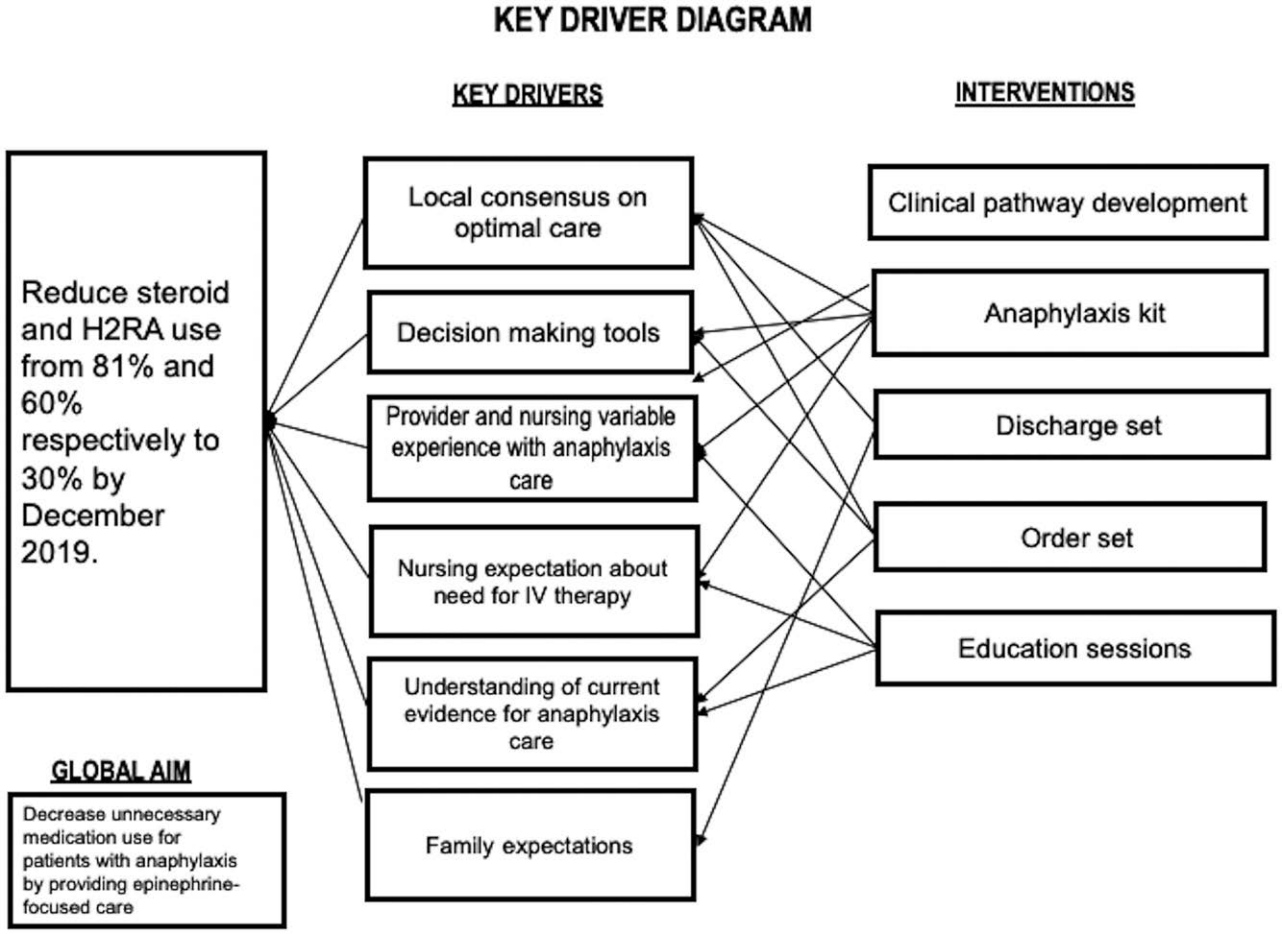
Key driver diagram.

**Fig. 2.**
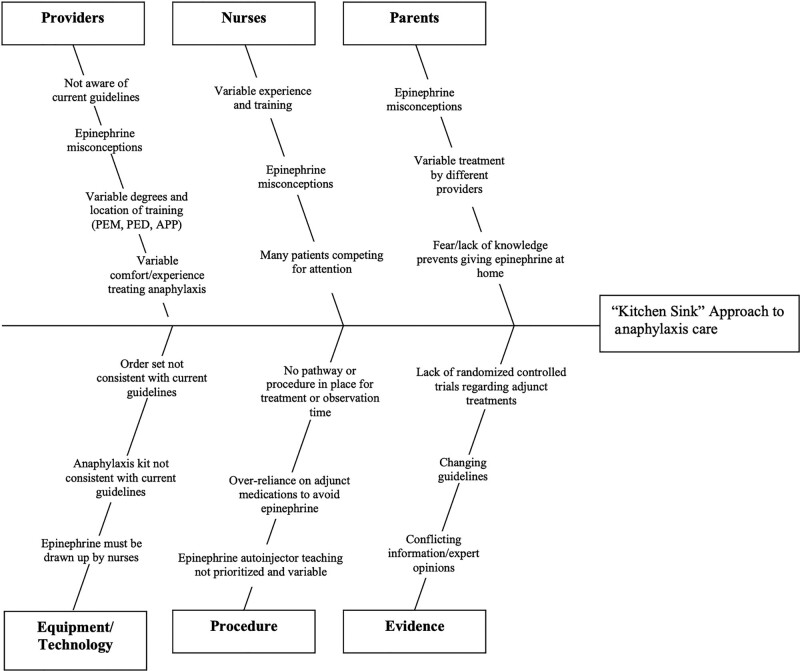
Ishikawa diagram for “kitchen sink” approach to anaphylaxis care.

After reviewing national anaphylaxis guidelines and local baseline data, the core improvement team established study goals and objectives. Next, we aimed to further understand the current state by creating process maps of anaphylaxis care for each acute care site. Finally, we used a driver diagram (Fig. 1) and an Ishikawa diagram (Fig. 2) to identify key drivers and barriers for the de-implementation of steroids and H2RAs.

The major key drivers identified were (1) providers’ and nurses’ variable experience with anaphylaxis recognition and treatment; (2) frequent use of IV steroids and IV H2RAs, which led to the presumed need for IV placement even before provider evaluation; (3) lack of a local consensus on optimal care; (4) decision-making tools, including EHR order sets and anaphylaxis kits, were reflective of current practice (both contained steroids and H2RA).

Next, a Kaizen event took place during which the QI team defined the ideal state of anaphylaxis care, selected interventions, and outlined implementation strategies. We could not identify national benchmarks for steroid and H2RAs use, so we utilized consensus and expert opinion to establish outcome targets. Finally, the QI team met monthly to discuss study data and review ongoing action items.

## INTERVENTIONS

### Intervention 1. Clinical Pathway Development and Dissemination (July 2018 to September 2019)

The first intervention was creating an anaphylaxis clinical pathway^11^ that further outlined the ideal future state of anaphylaxis care and aimed to standardize anaphylaxis care across all hospital settings (including acute care, inpatient, and ambulatory). National anaphylaxis guidelines informed the majority of the pathway content. We utilized local expert opinion when no published data were available. This document met the criteria for clinical pathway by local institutional review. The pathway document was easily assessable via the centralized pathway, internet, and intranet sites. The full pathway document is available in **Supplemental Digital Content 1,** which describes the anaphylaxis clinical pathway, http://links.lww.com/PQ9/A359.

The clinical pathway divided anaphylaxis treatments into first-line therapy (IM epinephrine), adjunct/symptomatic therapy (H1RA, bronchodilators, fluids, and racemic epinephrine), and second-line therapies (intravenous or oral steroids). The pathway document stated indications for each treatment. Specifically, the pathway recommended against routine use of H2RAs and steroids but advised using steroids for patients with severe anaphylaxis, concurrent asthma exacerbation, or those with airway concerns. The pathway also outlined (1) recommendations for ED observation, including early discharge criteria (less than 4 hours); (2) admission criteria; and (3) risk factors for biphasic reaction and severe or fatal anaphylaxis.

Dissemination (July 2019 to September 2019) included (1) presentation at mandatory educational meetings for both providers and nurses; (2) emails to nursing, providers, and pharmacy groups; and (3) information in provider and nursing newsletter. Nursing education also emphasized the recognition of signs and symptoms of anaphylaxis. In addition, providers completed an attestation survey confirming they reviewed the pathway.

### Intervention 2. Anaphylaxis Order Set (August 2019)

The existing EHR order set listed multiple medications for anaphylaxis and lacked any guidance or evidence-based prescribing cues. Therefore, the QI team implemented a new EHR order set to highlight epinephrine use and de-emphasize adjunct and second-line medications. The new order set also included indications for each treatment, monitoring parameters, and recommended observation time to guide decision-making. Finally, the order set included automated weight-based dosing for epinephrine so that providers could order essential pathway actions in a single step for most patients.

### Intervention 3. Anaphylaxis Medication Kit Revision (July 2019)

Our hospital system utilized standardized anaphylaxis medication kits. These kits include physically grouped medications in a portable container located in an easily accessible area within a medication dispensing system. The anaphylaxis kit could be brought to the bedside by a pharmacist or a nurse during initial evaluation and resuscitation, allowing for expedited medication administration. Before the study, anaphylaxis kits contained epinephrine, oral and IV steroids, H1RA and H2RAs, resulting in simultaneous administration of many of these medications. During this study, we revised anaphylaxis kits across the hospital system to be concordant with the newly developed anaphylaxis pathway. New kits no longer contained oral steroids and H2RAs and included copies of the anaphylaxis pathway. The goal of this change was to shift the focus away from combination therapy to epinephrine-only administration. However, providers could still order additional medications nonemergently via an anaphylaxis order set.

## MEASURES

### Outcome Measures

The primary outcome measure was the percentage of patients with primary ED/UC diagnosis of anaphylaxis who had a corticosteroid such as methylprednisolone, dexamethasone, prednisolone, or prednisone administered during their acute care visit. A second primary outcome measure was the percentage of patients with anaphylaxis given an H2RA (famotidine and ranitidine) during ED/UC visits.

### Process Measure

The process measure was the percentage of patients diagnosed with anaphylaxis who had a peripheral intravenous catheter (PIV) placed in ED/UC.

### Balancing Measure

Balancing measures included (1) length of stay (LOS) in the acute care site; (2) the percentage of patients who returned to an acute care site within 72 hours of initial visit; and (3) percent of patients admitted from ED/UC.

## ASSESSING IMPACT

### Data Collection Strategy

We collected and analyzed monthly data from EHR queries using ICD-10 codes for anaphylaxis (ICD-10 codes: T78.0, T78.2, T80.5, T88.6). At the beginning of the study, the QI team validated this measurement system by conducting manual chart reviews from a single-month report (35 charts) to ensure data accuracy in an automated report. In addition, the QI team manually extracted data monthly during the study period to track selected variables such as PIV insertion and epinephrine administration before ED/UC arrival. Finally, project leads reviewed all visits resulting in admission or return to ED/UC within 72 hours of the initial visit to identify the rationale for admission or return (specifically, to determine the frequency of suspected biphasic reaction in this group).

### Data Analysis

We used statistical process control charts (Shewhart charts) to analyze the outcome, process, and balancing measures.^12^ P charts displayed binomial variables, and Xbar-S charts displayed continuous variables with subgroups. In addition, we used Nelson’s rules to detect special cause variation in outcomes.^13^ Minitab Statistical Software Version 19 (State College, Pa.) was used to create control charts.

## RESULTS

**Fig. 3.**
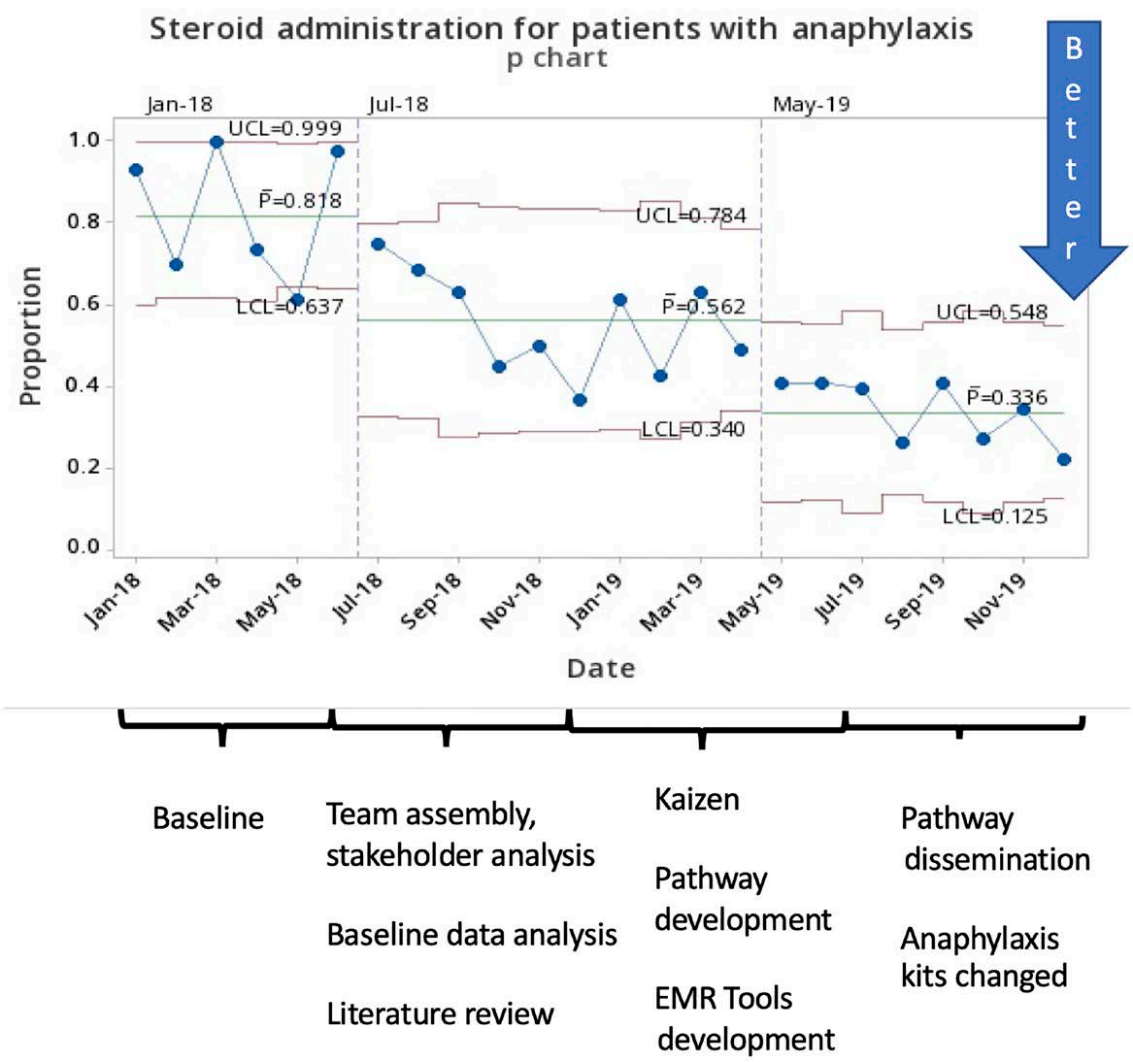
Steroids administration for patients with anaphylaxis.

**Fig. 4.**
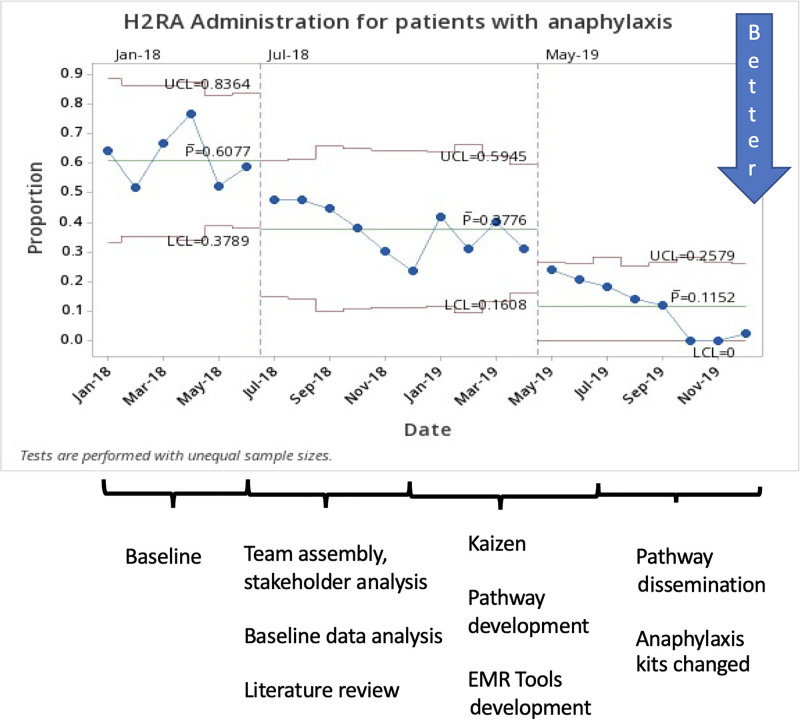
H2RA administration for patients with anaphylaxis.

The study included 870 patients from January 2018 to December 2019. There were 642 ED patients and 228 UC patients. Baseline data were collected from January 2018 to June 2018 (209 patients). The mean age was 9 years. Fifty-five percent of patients were male, and 45% were female. All patients in the study received epinephrine either in our acute care facility or from EMS, caregiver, or another facility. The P chart showed special cause variation (8 points on one side of the centerline) for the use of steroids (81%–33%) and H2RAs (60%–11%), demonstrating improvement (Figs. 3 and 4). At UC sites, steroid utilization decreased from 83% to 31%, whereas at emergency sites, steroid use decreased from 80% to 32%.

**Fig. 5.**
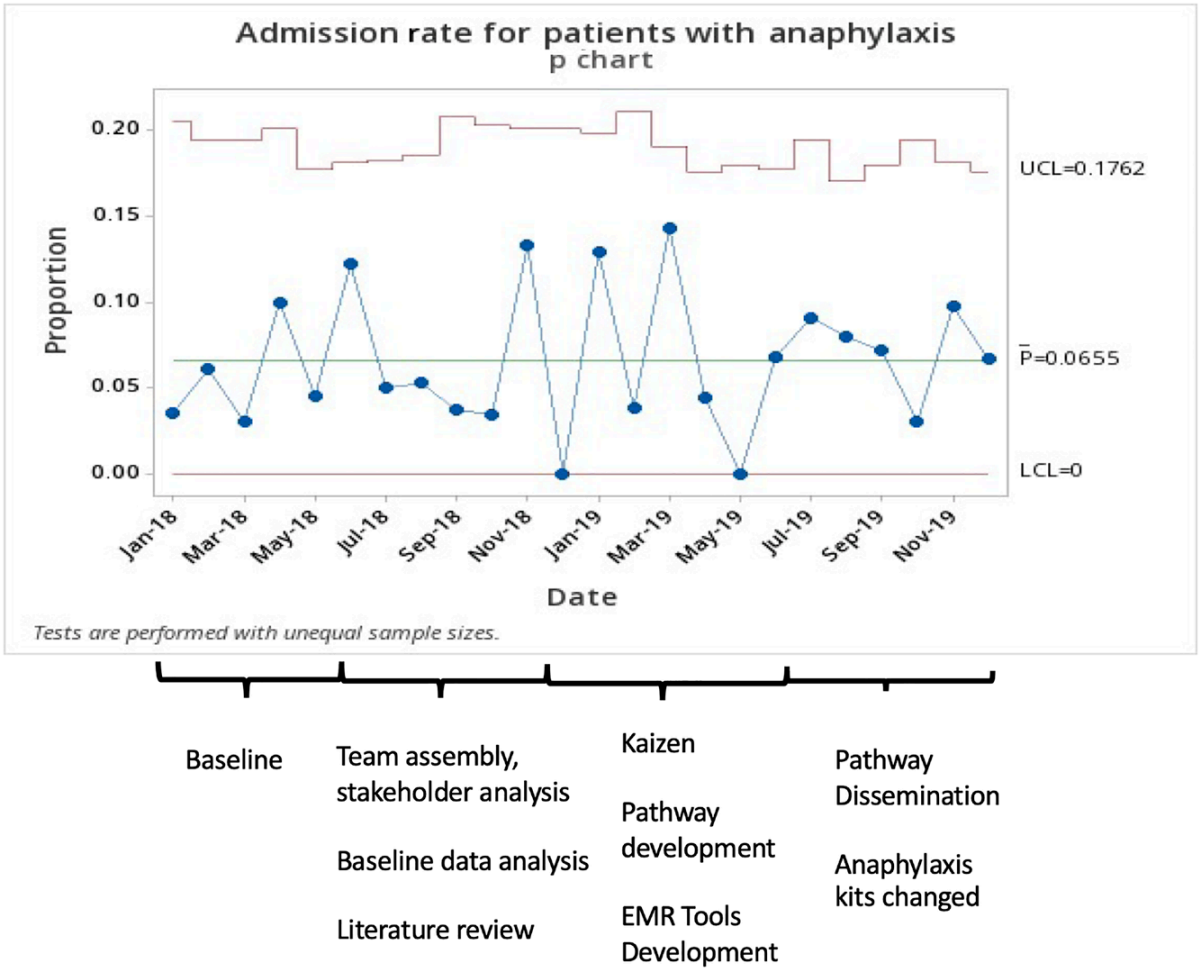
Admission rate for patients with anaphylaxis.

H2RA use decreased from 79% to 10% at UC sites and from 54% to 12% at emergency sites. IV placements decreased from 44% to 36%, although no special cause variation was detected on the p chart. LOS decreased from 6.2 to 5.0 hours (special cause variation detected, 6 points decrease in a row, and 4 out of 5 points more than one standard deviation from the centerline, **Supplemental Digital Content 2,** which describes ED/UC LOS, http://links.lww.com/PQ9/A359). There was no special cause variation in admission rate (6.7%–6.5%, Fig. 5) or return visits rate (4.3%–3.9%). We audited admissions and return visits from January 2019 to December 2019 (a total of 467 patients). During that time, 35 (7.5%) patients were admitted; 25 of 35 admitted patients (71%) received steroids during the initial ED/UC visit. During the same period, 22 (4.7%) patients returned to ED within 72 hours; 10 of 22 (45%) return visits received steroids during the initial ED/UC visit. Audits identified biphasic reactions in 8 of 467 patients (1.7%) during that time.

## DISCUSSION

This study demonstrated sustained de-implementation of corticosteroid and H2-receptor antagonist use in pediatric patients with anaphylaxis with the implementation of an epinephrine-focused clinical pathway. We achieved these results while decreasing ED/UC LOS and maintaining stable admission and 72-hour return rates.

The American Academy of Asthma, Allergy, and Immunology national anaphylaxis guidelines support targeted steroids and H2RA use in anaphylaxis.^5,6^ H2RAs do not prevent biphasic reaction,^6^ and there is inconsistent evidence of their positive effect on cutaneous symptoms.^14–16^ Therefore, in our clinical pathway, we did not give indications for H2RAs use. Our postintervention use of H2RAs was 11% without a negative impact on the LOS, return visits, or admissions. However, we did not evaluate how decreased H2RA use affected the severity or duration of cutaneous symptoms.

Traditionally, steroids are given to decrease the incidence of biphasic reaction, despite limited evidence supporting this rationale for use.^7–9^ The use and dose of steroids for anaphylaxis were extrapolated initially from those used for acute asthma exacerbations.^17^ We audited visits resulting in admission or unplanned ED returns and found biphasic reaction to be an infrequent cause for admissions and return ED visits. The reported frequency of biphasic reactions in anaphylaxis varies, with estimates as high as 20%. Still, more recent studies estimate 4%–5% when using the National Institute of Allergy and Infectious Disease and Food Allergy and Anaphylaxis Network criteria.^6,8^ In our study population, 1.7% of patients seen in the postintervention period were admitted or returned to the ED/UC with concern for biphasic reaction. Admission and unplanned ED visits did not increase as steroid use decreased. In addition, the majority of admitted and returning patients did receive steroids in ED/UC. Outlining criteria for steroid use helps identify patients that benefit the most from this intervention, such as those with severe anaphylaxis and concomitant asthma exacerbation. De-emphasizing steroids in order sets and anaphylaxis kits allowed de-implementation of universal use, ensured buy-in from acute care providers and staff, and contributed to the sustainability of this practice change.

About a third of our study population received care in an UC setting. UC sites had similar baseline utilization of steroids (83% in UC and 80% in ED) and higher baseline H2RAs (79% in UC and 54% in ED). Postintervention utilization for both steroids and H2RAs was similar in both UC and ED settings (32% in UC and 31% in ED for steroids; 12% in UC and 12% in ED for H2RAs). Similar outcomes for UC versus emergency care setting is likely related to our practice environment. Study interventions, including EMR changes, anaphylaxis kits changes, and dissemination efforts for providers and staff, were implemented similarly at all sites. In addition, most of our providers routinely rotate between different sites. Thus, providers who typically staff UC sites also rotate through ED sites and vice versa. Therefore, individual practice and the availability of tools for guideline adherence are shared between practice settings.

We used ED/UC LOS as a balancing measure because decreased steroid use might increase observation times. Rather than increase, we saw a decrease in LOS by 20% during the project period, which was unexpected. Our clinical pathway recommends an observation time of 4 hours for most patients, prolonged observation for severe anaphylaxis, or those at risk for a biphasic reaction. It includes criteria for early discharge (under 4 hours). Standardizing recommended observation times and clear admission criteria likely contributed to more efficient care without an increase in unplanned visits or admissions. Similar outcomes of standardizing observation times without adverse effects on return rates were found by Farbman et al^4^ and Lee et al^18^ at two other major pediatric institutions. However, these studies did not address steroid and H2RA use recommendations.

### Barriers

The QI team encountered barriers to solution implementation. First, the pathway team had to ensure buy-in from a multidisciplinary group of stakeholders, including those representing inpatient and ambulatory clinical areas. We performed a thorough stakeholder analysis and met with individuals to describe project aims and address concerns. In addition, we gave ample opportunity for providers and staff to ask questions about pathway guidance and EMR tools during in-person educational sessions and via email. We also disseminated admission and return rate data to address concerns about unintended consequences of pathway guidance. As a result, we did not encounter significant resistance with steroid and H2RA guidance from ED/UC providers and staff once we outlined and disseminated utilization criteria. At the same time, guidance on observation time generated more significant debate, especially regarding the observation in UC settings and requirements for interfacility transfer.

Another barrier was the lack of a formal process to change medication kits. Since anaphylaxis kits were standardized throughout the hospital system, we had to involve additional stakeholders. In addition, there was a robust discussion about the optimal content for these medication kits. As a result, we worked with pharmacy administration and representatives from different clinical areas to develop a process to update medication kits and optimize kit content.

### Limitations

Our study had several limitations. First, this study took place at an institution that has an established pathway program. Therefore, we were fortunate to have a culture of evidence-based guideline use and utilize existing pathway development and implementation resources. However, general concepts behind our interventions revolve around care standardization and implementation and may not need extensive resources. Second, our clinical pathway outlined criteria for steroid use, and overall use decreased dramatically. However, we did not specifically audit data to determine to what extent steroids used was concordant with pathway recommendations (right medication for the right patient). Next, we only included patients that had a billing diagnosis of anaphylaxis. The epinephrine utilization rate in our study population was 100%, likely because providers associate anaphylaxis diagnosis with giving epinephrine. It is possible that some patients that experienced anaphylaxis did not receive epinephrine and had a different billing diagnosis despite meeting clinical criteria. Therefore, we cannot comment on how our interventions impacted overall epinephrine administration rates in those meeting clinical criteria. Last, we noted a decrease in steroid use even before the pathway was formally published on the pathway website. Several providers participated in pathway development and generally indicated favorable attitudes among ED/UC providers to decreasing steroid and H2RT use. There was a general awareness of this pathway development. Several early adopters changed their practice based on national recommendations even before the formal local recommendations became available.

## CONCLUSION

This study demonstrated safe, sustainable de-implementation of systemic steroids, and H2RAs in pediatric patients with anaphylaxis using QI methodology.

## DISCLOSURE

The authors have no financial interest to declare in relation to the content of this article.

## ACKNOWLEDGMENTS

Leigh Anne Bakel, MD, MSc, Director of the MSc Pathway Program. Pediatric Emergency Medicine Team: Ryan Caltagirone, MD, Cortney Braund, MD, Hannah Griffin, PNP, Maya Haaz, MD, Joni MacKenzie, PNP, Sarah Moultrie, PNP, Kevin Poel, Pharm D, Hayley Ross, MD, Lane Shirley, MD, and Amanda Stump, PNP, for the assistance with this study.

This work was accepted for poster presentation at the Pediatric Academic Societies Meeting, Philadelphia, PA. May 1–5, 2020. [Conference canceled due to COVID-19-related travel restrictions]

## Supplementary Material


