# Supplementary material for: Minimizing the “Kitchen Sink” Approach: De-implementation of Unnecessary Care for Patients with Anaphylaxis in a Pediatric Emergency Department
Source: Pediatr Qual Saf. 2022 Mar 30;7(2):e535. doi: 10.1097/pq9.0000000000000535 (PMC8970089; doi:10.1097/pq9.0000000000000535)
Supplement: Supplementary file 1 [file pqs-7-e535-s001.pdf]

Children's Hospital Colorado

ANAPHYLAXIS

ALGORITHM

***Signs and Symptoms of Anaphylaxis[5]**

To meet definition for anaphylaxis, must meet #1, #2, or #3 below:

1. Acute onset skin and/or mucosal involvement AND at least one of the following:
- Respiratory compromise
- Reduced blood pressure or symptoms of end-organ dysfunction

2. Two or more of the following that occur rapidly:
- Involvement of the skin/mucosal tissue
- Respiratory compromise
- Reduced blood pressure, or associated symptoms
- And/or persistent gastrointestinal symptoms

3. Hypotension after exposure to known allergen

Inclusion Criteria
- Signs and Symptoms of Anaphylaxis*
- Received Epinephrine prior to arrival for presumed anaphylaxis
- Exposure to known allergen
- Age 3 months to 21 years

Exclusion Criteria
- Symptoms clearly attributed to other causes
- In the OR, ICU, or Allergy Clinic
- Receiving blood transfusion
- CCBD anticipated anaphylactic infusions reactions with a provider at the bedside, reversal medications preordered in Epic and ready at the bedside, and following specific medication reaction guidelines as outlined by the pharmaceutical company

Does the patient currently have signs/ symptoms of anaphylaxis*?

NO → Consider lower Epi threshold for patients with **red flags. Epi given?
- YES → Stop offending agent/infusion
- NO → Did patient get epi prior to arrival?
  - YES → Rapid Assessment
  - NO → See Disposition page 8

YES ↓

Stop offending agent/infusion

Give IM Epinephrine
- Epinephrine(Epi) 0.01mg/kg/dose with max dose of 0.5mg of the 1mg/ml concentration (administered IM)
- Repeat every 5-15 minutes as needed
- Give Epinephrine Auto Injector if appropriate for care setting

Rapid Assessment:
- Position patient supine
- Oxygen as needed for hypoxemia
- Full CR monitoring with blood pressure cycle every 5 mins
- Consider higher level of care (if in Urgent Care or Clinic, transfer by ambulance)

Adjunct Therapy For Symptomatic Care:
- Albuterol for wheezing
- Racemic Epinephrine for stridor
- IV access
- Normal Saline bolus for hypotension
- Anti-emetic only with concern for aspiration, not routinely recommended

Is the patient improving? NO → Severe Anaphylaxis; YES → Disposition

Severe Anaphylaxis:
- Patient received 3 or more doses of Epi and/or has persistent cardiorespiratory symptoms
- Follow PALS Guidelines and initiate IV fluid resuscitation if patient is in shock
- Start Epinephrine drip. Start at 0.1mcg/kg/min and titrate (up to 1mcg/kg/min). Stop IM epinephrine once epinephrine drip initiated.
- Anticipate difficult airway
- **See Pages 5, 6 & 7** for additional clinical management and therapeutic guidelines
- Disposition PICU

!

Inpatient Considerations
- **Code Blue** if patient has respiratory compromise or hypotension.
- **RRT** for all other patients

****Patients with the following red flags are at higher risk for severe, prolonged and/or biphasic anaphylaxis or death:**
- History biphasic or delayed reaction
- Received more than one dose epinephrine with current episode
- Non-verbal
- Difficult airway
- Current asthma exacerbation
- Significant co-morbidities
- Delayed Epi administration (30 minutes or greater from onset of symptoms)
- Facial or airway swelling with current episode

Second Line Therapy:

Glucocorticoid steroids and H2 antagonists do not have a proven benefit in Anaphylaxis
- Consider PO Cetirizine/IV Diphenhydramine for itching.
- Consider steroids as a second line therapy with asthma, severe anaphylaxis, or other airway concerns

Disposition:

4 hours of observation from Epi administration is recommended. Some patients may be eligible for earlier discharge. **See page 8** for details

Early Discharge Criteria:
- Serious symptoms have resolved (rash may persist) and patient feels well.
- Epinephrine auto-injector in hand or available at home.
- Epinephrine auto-injector teaching has been completed
- Patient does not have any **Red Flags

Consider Admission or Prolonged Observation With:
- History of severe, delayed or biphasic reaction
- Required additional IM epinephrine during observation period

TABLE OF CONTENTS

[Algorithm]
[Target Population]
[Background | Definitions]
[Initial Evaluation]
[Clinical Management]
[Therapeutics]
[Disposition]
[Parent | Caregiver Education]
[References]
[Clinical Improvement Team]

TARGET POPULATION

Inclusion Criteria

Patients with:

- Signs and Symptoms of Anaphylaxis
    - To meet definition for anaphylaxis, must meet #1, #2, or #3 below:
        - 1. Acute onset of a reaction (minutes to hours) with involvement of the skin and/or mucosal tissue AND at least one of the following
            - Respiratory compromise
            - Reduced blood pressure or symptoms of end-organ dysfunction
        - 2. Two or more of the following that occur rapidly after exposure to a likely allergen:
            - Involvement of the skin/mucosal tissue
            - Respiratory compromise
            - Reduced blood pressure, or associated symptoms
            - And/or persistent gastrointestinal symptoms
        - 3. Hypotension after exposure to a known allergen
- Received Epinephrine prior to arrival for presumed anaphylaxis
- Exposure to known allergen
- Age 3 months to 21 years

Exclusion Criteria

- Age less than 3 months
- Receiving blood transfusion
- Symptoms clearly attributed to other causes
- Patient is physically present in the OR, ICU or allergy clinic (receiving diagnostic evaluation and/or treatment).
- CCBD anticipated anaphylactic infusions reactions with a provider at the bedside, reversal medications preordered in Epic and ready at the bedside, and following specific medication reaction guidelines as outlined by the pharmaceutical company

Please note: There is no absolute contraindication to administration of epinephrine for anaphylaxis.

In the cardiac population, risk-to-benefit ratio needs to be assessed with care, but usually favors the administration of epinephrine.[3]

BACKGROUND | DEFINITIONS

Anaphylaxis Background:

Anaphylaxis is a defined as an allergic reaction that is rapid in onset and may cause death[1]. Systemic symptoms result from sudden release of multiple mediators, with broad classification of anaphylaxis being subdivided into immunological causes (i.e. those mediated by immunoglobulin E (IgE)), non-immunological (i.e. exercise induced anaphylaxis) and idiopathic causes.[2] Anaphylaxis is thought to be increasing in prevalence with the most common triggers being, in decreasing order of incidence, food, drug/biologics, insect stings, idiopathic, exercise induced and other allergens[3,4].

While there is no universal agreement on its definition or criteria for diagnosis, the most commonly cited diagnostic criteria are from NIAID/FAAN (National Institute of Allergy and Infectious Disease/Food Allergy and Anaphylaxis Network Symposium)[1]. There are three different clinical criteria as indicated above to indicate when anaphylaxis is highly likely[1]. These criteria have been shown to accurately diagnose anaphylaxis in an emergency department setting with a sensitivity of 96.7% and a specificity of 82.4%[5]. The NIAID/FAAN guidelines note, however, that there will be patients who do not fulfill these criteria in whom it would be appropriate to initiate therapy with epinephrine[1].

Intramuscular Epinephrine is first line treatment for anaphylaxis and delays in administration have been associated in fatalities[3]. It is also essential to remove the inciting allergen, i.e. stop a medication infusion, if applicable. Addition initial management includes ‘ABC’s’ as detailed in the management section, including oxygen supplementation if needed and placing the patient in the supine position. Antihistamine and corticosteroids are never initial therapy for anaphylaxis and are considered optional or adjunctive[3].

Definitions:

Anaphylaxis: An allergic reaction that is rapid in onset and may cause death[1]

Bi-Phasic Reaction: Late-phase reaction that can occur 1-72 hours after remission of initial attack (reported in 1-23% of patients)[3].

Severe/Protracted Anaphylaxis: Severe anaphylactic reaction that may last between 24-36 hours despite aggressive treatment.[3]

Uni-phasic Anaphylaxis: isolated reaction producing signs and symptoms within minutes (typically within 30 minutes) of exposure to an offending stimulus.[3]

INITIAL EVALUATION

- Patients present with actual or reported signs/symptoms suggestive of anaphylaxis (See: Inclusion criteria and Background)
  - Administer epinephrine prior to detailed history and physical
  - Obtain rapid assessment while preparing epinephrine
  - If in ED or Urgent care, Triage ESI (Emergency Severity Index) minimum level 2. Otherwise, activate emergency protocol most appropriate for your care area (ie, code, RRT or dial 911).
  - Notify provider
  - Notify respiratory therapist if: respiratory symptoms are present, history includes asthma, or patient has known difficult airway
- Rapid assessment (by nurse, provider)
  - Vital signs including pulse, respiratory rate, blood pressure, oxygen saturation
  - Rapid airway assessment including auscultate for wheezing, stridor, decreased aeration
  - Rapid circulatory assessment including capillary refill, pulses, color
  - Rapid neurological assessment including AVPU (alert, verbal, pain, unresponsive)

- Focused history including:
  - Exposure to known, suspected, or common allergen prior to onset of symptoms
  - Administration of epinephrine for suspected anaphylaxis prior to arrival, and response
  - Timing of onset of symptoms
  - Epi delayed from onset of symptoms (30 minutes or greater)
  - History biphasic or delayed reaction
  - Unable to describe symptoms related to developmental delay, nonverbal, or young age
  - Difficult airway
  - Current asthma exacerbation
  - Significant co-morbidities, including asthma (even if no exacerbation)

CLINICAL MANAGEMENT

First-Line Therapy:

Epinephrine:

If drawing up epinephrine from 1mg/mL vial:

- Epinephrine 0.01mg/kg/dose with maximum dose of 0.5mg of the 1mg/ml concentration administered intramuscularly (IM) into lateral thigh, repeat every 5 minutes as needed

OR

If using Epinephrine Auto Injector (EAI):

- 7.5-15kg=0.1mg
- 15.1-30kg=0.15mg
- >30kg=0.3mg
- Patients less than in the 7.5kg range should have their patient specific dose drawn up from a vial if possible.

Adjunct Therapies:

Positioning

- Patient should be positioned supine unless there is significant respiratory distress, then the patient should sit in their position of comfort. Please note fatalities have occurred with rapid changes from supine to a non-supine position during anaphylaxis.

Hypoxemia and/or airway concerns

- Provide oxygen as needed for hypoxemia
- Provide short-acting beta-agonist for evidence of lower airway obstruction (wheezing, increased work of breathing, prolonged expiration, etc.)
- Provide Racemic Epinephrine for stridor

Cardiovascular concerns

- Provide boluses of intravenous (IV) fluids of 20mL/kg of normal saline for hypotension. If patient does not respond after 3 doses of epinephrine IM or has signs of impending cardiovascular collapse start epinephrine drip. Start at 0.1-1mcg/kg/min and titrate

- Consider administration of glucagon if patient is on a beta blocker.

Concern for aspiration:

- If concern for aspiration, may provide anti-emetic such as ondansetron.
- Routine administration of anti-emetics is not recommended as it is beneficial for patients to vomit offending agent.

Severe Anaphylaxis

Patients who require 3 or more doses of epinephrine or have severe, persistent cardiorespiratory symptoms, should be considered to have severe anaphylaxis. These patients require immediate escalation of care which may include:

- Call a code or initiate emergency response if outside the Emergency Department and if not already done
- Start an IV epinephrine infusion.[3]
    - Begin infusion at 0.1mcg/kg/min and titrate up
- Nebulized racemic epinephrine should not be used as first line but may be used as an adjunct if airway edema is present.
- Early intubation is recommended. Anticipate a difficult airway.
    - Consult Anesthesia
    - A smaller than usual endotracheal tube may be necessary
    - Consider Ketamine as sedation agent for rapid sequence intubation (RSI)
    - Avoid paralytics if possible.
- If shock, follow PALS guidelines
    - Place 2nd IV
    - Initiate aggressive IV fluid resuscitation
    - Consider additional pressor support if persistent hypotension (norepinephrine or vasopressin)
- If patient is on a beta-blocker, administer Glucagon[3]
- Consider ECMO
- Once stabilized, admit to ICU.

Inpatient Management Considerations

Escalation of care for inpatients:

- A CODE BLUE should be called for: patients with any evidence of cardiorespiratory compromise (oral swelling [tongue and/or uvula, but not lips alone], airway swelling, stridor, hypoxia, hypotension, poor perfusion, etc).
- An RRT should be called for all other instances of anaphylaxis/administration of IM epinephrine
- If there is uncertainty as to severity of symptoms OR a provider (MD/DO/PA/NP) is not available to immediately come to the bedside to assess patient, then a Code Blue should be called.

Inpatient Discharge Criteria

- Auto-injector in hand and teaching complete (including allergy action plan). Please note that due to a worldwide shortage it may sometimes be difficult to obtain; discuss with Allergy and/or inpatient pharmacy if unable to locate at our Walgreen's or an easily accessible outpatient pharmacy.
- Allergy follow-up recommended (referral should be placed by PCP as usual)
- Resolution of symptoms (with exception of rash/pruritis) and vitals normal for age for at least 12 hours post-epinephrine

Outpatient Management Considerations

If a patient presents with signs or symptoms concerning for anaphylaxis, the health care provider should be notified immediately to perform an evaluation. There are NO absolute contraindications to epinephrine in the setting of anaphylaxis.

After epinephrine is provided, continue following the algorithm for monitoring. Based on clinical symptoms, follow your clinic's protocol for activating an emergency response or calling 911. All patients require a period of observation after receipt of epinephrine. Some patients may be appropriate for monitoring in the outpatient clinic if adequate space and resources are available.

THERAPEUTICS

- Epinephrine IM
  - Dose: 0.01mL/kg (max dose of 0.5mL) using 1mg/mL concentration
  - Route: IM: preferred site lateral thigh
  - Frequency every 5-15 minutes as needed
- Epinephrine IV Drip
  - Dose: 0.1-1mcg/kg/min (start at 0.1mcg/kg/min and titrate up)
  - Concentration: 10mcg/mL, 20mcg/mL and 100mcg/mL (central line only)
    - 0.6mg (0.6mL) of 1:1000 epi in 29.4mL of NS to yield a 20mcg/mL, 30mL syringe
  - Route: IV
  - Frequency: Continuous
- Norepinephrine IV Drip: For warm shock
  - Dose: 0.05-0.1mcg/kg/min (4-12mcg/min in Adults)
  - 32mcg/mL premixed bag Route: IV
  - Frequency: Continuous
- Albuterol
  - Dose: <20kg=2.5mg; ≥20kg=5mg
  - Route: inhalation
  - Frequency: as needed, for anaphylaxis per provider instruction
- Racemic Epinephrine (2.25% solution)
  - Dose: 0.05 mL/kg (maximum dose: 0.5 mL) diluted in 2 to 3 mL Normal Saline
  - Route: inhalation
  - Frequency As needed, for anaphylaxis per provider instruction
- IV Fluids:
  - Dose: 20ml/kg
  - Route: IV
  - Frequency: Once
- Ketamine for sedation during Rapid Sequence Intubation
  - Dose: 2mg/kg. Max 100mg
  - Route: IV
  - Frequency

- Glucagon: If patient is not responding to epinephrine **and the patient is on beta-blockers**, consider administration of glucagon
  - Dose: Initial: 20 to 30 mcg/kg (maximum: 1 mg), infused slowly; followed by an infusion of 5 to 15 mcg/minute; titrate the infusion rate to achieve an adequate clinical response[3]
  - Route: IV
  - Frequency:
- Zofran: For concern for aspiration only. Not for routine administration
  - Dose: 0.1mg/kg max of 4mg
  - Route: IV/PO
  - Frequency: once

Second-Line Therapies:

Antihistamines: There is no direct evidence to support their use in the treatment of anaphylaxis. H1 antihistamines may be helpful for symptomatic treatment of itching, hives, rhinorrhea, sneezing based on data extrapolated from other allergic conditions. However, they do not prevent or treat upper airway obstruction or hypotension.[1,2,3,4]

H1-blockers:

- Cetirizine, 2nd generation, non-sedating antihistamine. Preferred to sedating antihistamine.
  - Doses: <6 months: 1.25mg; 6-24 months: 2.5mg; 2-5 years old: 5mg; >5 years old: 10 mg
  - Route: PO
  - Interval: Once daily. May be increased to twice daily for max of 5mg daily for <2yo.
- Diphenhydramine, 1st generation antihistamine. If patient is unable to take oral medication or non-sedating anti-histamine is not available, can use diphenhydramine
  - Dose: 1mg/kg/dose, max 50 mg
  - Route: IV/PO
  - Interval: every 6 hours.

H2-blockers: use not supported by randomized, placebo-controlled trials[2] IV cimetidine should not be used, as it can increase hypotention[3])

- Ranitidine, H2-blocker
  - Dose: IV=1mg/kg/dose, max 50 mg; PO=2mg/kg max of 300mg
  - Route: IV/PO
  - Interval: IV every 6-8 hours; PO every 12 hours

Corticosteroids: have no role in the acute management of anaphylaxis.[1] The prior reported evidence that they decrease biphasic or prolonged reactions is not supported by strong evidence.[1,2,3,6] Consider using glucocorticoids in those with evidence of lower airway obstruction, asthma, rebound anaphylaxis or severe anaphylaxis.

- Methylprednisolone or steroid equivalent
  - Dose: 2mg/kg (max 125mg) x1
  - Route: IVIM
- Prednisolone/Prednisone
  - Dose: 2mg/kg max 60mg x1
  - Route: PO

- Do not continue steroids if patient recovers.
- Consider continuing steroid if patient is hospitalized in the pediatric ICU or has frequent idiopathic anaphylaxis (at least two episodes in the preceding two months or at least six episodes in the preceding year)

DISPOSITION:

- Most patients will require 4 or more hours of observation post epinephrine administration.
- Some patients who had mild symptoms that resolved quickly with administration of epinephrine and remain asymptomatic, may be eligible for discharge earlier.

Early Discharge Criteria:

1) No respiratory symptoms with this episode.
2) Symptoms resolved after a single dose of IM epinephrine.
3) Serious symptoms have resolved (rash may persist) and patient feels well.
4) Epi auto-injector in hand or available at home.
5) Epinephrine auto-injector teaching has been completed.
6) Lives in close proximity to a healthcare facility.
7) No red flags in history or associated with this event.

- Patients who present with exposure to a known allergen but who did not have symptoms, have no red flags, and did not require epinephrine may not require an observation period.
- Any patient who presented with cardiac or respiratory symptoms or other red flags (history of biphasic or delayed reaction, required >1 does of epinephrine, is unable to verbalize symptoms due to age or developmental delay, has a current asthma exacerbation or history of significant asthma, >30min delay in administration of epinephrine, a known difficult airway, or significant co-morbidities) should be observed for a minimum of 4-6 hours.
- Hospital admission or prolonged observation (12-24 hours) should be considered for patients with a history of severe, delayed or biphasic reaction.   Patients with severe anaphylaxis should be admitted to the ICU.

Discharge Therapeutics:

Epinephrine Auto Injectors: Auto-injectors are typically available in pre-fixed doses, 0.15 and 0.30 mg. In children, the selection of the dose should be guided by the dose recommended for anaphylaxis (0.01 mg/kg for children, maximum of 0.30 mg). Given the safety profile of epinephrine, and considering underdoing may not effectively treat anaphylaxis, giving a dose via auto injector that is slightly above the ideal dose appears to be a better option than giving a dose that is below the recommended dose.[3]

- >30 kg use 0.3mg/adult dose
- 15.1kg - 30kg use 0.15mg/pediatric dose
- 7.5kg - 15kg use 0.1mg/infant dose
- Patients <7.5kg can be taken on a case by case basis and provider can assess risk/benefit of giving 0.1mg auto-injector or teaching family to draw up dose from vial
- Please note 0.1mg infant dose auto injector is not universally available

If swelling, hives, pruritus still present, consider as needed antihistamines.

If anaphylaxis has resolved, it is not necessary to give discharge recommendations for antihistamine or steroid prescription.[1,3]

PARENT | CAREGIVER EDUCATION

Epinephrine Auto Injector (EAI) prescription and training, a written anaphylaxis action plan, education on the plan and recognition of anaphylactic symptoms, and a recommendation to obtain medical identification jewelry, should all be provided[2,3]. Review of these items is important due to the following:

- Many patients do not fill Epinephrine Auto Injector prescription, and even fewer have allergy follow-up in the year following an emergency department visit for anaphylaxis.7
- Caregivers are not always able to correctly demonstrate the device, after a single one-to-one demonstration8, so re-demonstration even in a patient with a previous prescription for an EAI is beneficial.[9]
- Many patients need more than one dose of epinephrine, so patient should be given prescriptions for “dual packs” or “two packs” and instructed to keep the pair together. Thus prescribing multiple “two (or dual) packs” is recommended (patients should have dual packs at home as well as school/daycare, etc). [3]
- Additionally, the patient should be encouraged to follow-up with their primary care provider or allergy provider who manages their allergies. If trouble obtaining an EAI, one of these providers should be able to assist. If this is a new problem, suggest evaluation to allergist.

REFERENCES

1. Sampson HA, Muñoz-Furlong A, Campbell RL, et al. Second symposium on the definition and management of anaphylaxis: summary report Second National Institute of Allergy and Infectious Disease/Food Allergy and Anaphylaxis Network symposium. J Allergy Clin Immunol. 2006;117:391e397. IIIb.
2. Simons FE. Anaphylaxis. J Allergy Clin Immunol. 2010; 125(2 suppl 2): S161-81.
3. Lieberman et al. Anaphylaxis-A Practice Parameter Update 2015. Ann Allergy Asthma Immunol 115 (2015) 341-384.
4. Golden D. Patterns of anaphylaxis: acute and late phase features of allergic reactions. In: Anaphylaxis. Novartis Foundation, 2004.
5. Campbell RL, Hagan JB, Manivannan V, et al. Evaluation of National Institute of Allergy and Infectious Diseases/Food Allergy and Anaphylaxis Network criteria for the diagnosis of anaphylaxis in emergency department patients. J Allergy Clin Immunol. 2012;129:748e752.
6. Choo KJL, Simons E, Sheikh A. Glucocorticoids for the treatment of anaphylaxis: Cochrane systematic review. Oxford, UK2010:1205-11.
7. Motosue MS, Bellolio MF, Van Houten HK, Shah ND, Bellamkonda VR, Nestler DM, et al. Temporal trends in epinephrine dispensing and allergy/immunology follow-up among emergency department anaphylaxis patients in the United States, 2005–2014. J Allergy Clin Immunol Pract. 2017;5(5):1272–9 e1
8. Brown J, Tuthill D, Alfaham M, Spear E. A randomized maternal evaluation of epinephrine autoinjection devices. Pediatr Allergy Immunol 2013;24(2):173–177.
9. Fleischer, DM, Perry TT, Atkins D. Allergic Reactions to Foods in Preschool-Aged Children in a Prospective Observational Food Allergy Study. Pediatrics 2012; 130 (1): e25.

CLINICAL IMPROVEMENT TEAM MEMBERS

Laura Crouse, NP Emergency Medicine
Irina Topoz, MD | Emergency Medicine
Maureen Bauer, MD | Allergist
Kirstin Carel, MD | Allergist
David Fleischer, MD | Allergist
Hannah Duffey, MD | Allergy Fellow
Julia Freeman, MD | Emergency Medicine
Maya Haasz, MD | Emergency Medicine
Emily Albrecht, MD | Hospitalist
Alison Duggar, MD| Hospitalist
Cristin Fritz, MD | Hospital Medicine Fellow
Cinnamon Dixon, MD | Pediatrician
Hilary Stempel, MD | Pediatrician
Jennifer Griffin, PNP | Oncology
Kevin Poel, PharmD | Clinical Pharmacist
Kaleigh Ogawa, RN| Clinical Effectiveness
Mary Smith, RN| Emergency Department/Urgent Care
Beth Carroll, RN| Emergency Department/Urgent Care

APPROVED BY

Clinical Care Guideline and Measures Review Committee – 04/15/2019
Medication Safety Committee – June 2019
Pharmacy & Therapeutics Committee – 05/02/2019

**MANUAL/DEPARTMENT**	Clinical Pathways/Quality
**ORIGINATION DATE**	04/15/2019
**LAST DATE OF REVIEW OR REVISION**	9/11/2019
**COLORADO SPRINGS REVIEW BY**	Michael DiStefano, MD<br>Chief Medical Officer, Children's Hospital Colorado-Colorado Springs
**APPROVED BY**	Lalit Bajaj, MD, MPH<br>Medical Director, Clinical Effectiveness

REVIEW | REVISION SCHEDULE

Scheduled for full review on September 11, 2023

Clinical pathways are intended for informational purposes only. They are current at the date of publication and are reviewed on a regular basis to align with the best available evidence. Some information and links may not be available to external viewers. External viewers are encouraged to consult other available sources if needed to confirm and supplement the content presented in the clinical pathways. Clinical pathways are not intended to take the place of a physician's or other health care provider's advice, and is not intended to diagnose, treat, cure or prevent any disease or other medical condition. The information should not be used in place of a visit, call, consultation or advice of a physician or other health care provider. Furthermore, the information is provided for use solely at your own risk. CHCO accepts no liability for the content, or for the consequences of any actions taken on the basis of the information provided. The information provided to you and the actions taken thereof are provided on an "as is" basis without any warranty of any kind, express or implied, from CHCO. CHCO declares no affiliation, sponsorship, nor any partnerships with any listed organization, or its respective directors, officers, employees, agents, contractors, affiliates, and representatives.

Children's Hospital Colorado

Children's Hospital Colorado • Anschutz Medical Campus • 13123 East 16th Avenue • Aurora, CO 80045 • 720-777-1234 • childrenscolorado.org

Discrimination is Against the Law. Children's Hospital Colorado complies with applicable Federal civil rights laws and does not discriminate on the basis of race, color, national origin, age, disability, or sex. Children's Hospital Colorado does not exclude people or treat them differently because of race, color, national origin, age, disability, or sex.

Children's Hospital Colorado provides free aids and services to people with disabilities to communicate effectively with us, such as: Qualified sign language interpreters, written information in other formats (large print, audio, accessible electronic formats, other formats). Children's Hospital Colorado provides free language services to people whose primary language is not English, such as: Qualified interpreters, information written in other languages.

If you need these services, contact the Medical Interpreters Department at 720.777.9800.

If you believe that Children's Hospital Colorado has failed to provide these services or discriminated in another way on the basis of race, color, national origin, age, disability, or sex, you can file a grievance with: Corporate Compliance Officer, 13123 E 16th Avenue, B450, Aurora, Colorado 80045, Phone: 720.777.1234, Fax: 720.777.7257, corporate.compliance@childrenscolorado.org. You can file a grievance in person or by mail, fax, or email. If you need help filing a grievance, the Corporate Compliance Officer is available to help you.

You can also file a civil rights complaint with the U.S. Department of Health and Human Services, Office for Civil Rights, electronically through the Office for Civil Rights Complaint Portal, available at ocrportal.hhs.gov/ocr/portal/lobby.jsf, or by mail or phone at: U.S. Department of Health and Human Services 200 Independence Avenue, SW Room 509F, HHH Building Washington, D.C. 20201 1-800-368-1019, 800-537-7697 (TDD) Complaint forms are available at www.hhs.gov/ocr/office/file/index.html.

Children's Hospital Colorado complies with applicable Federal civil rights laws and does not discriminate on the basis of race, color, national origin, age, disability, or sex.

ATENCIÓN: si habla español, tiene a su disposición servicios gratuitos de asistencia lingüística. Llame al 1-720-777-9800.

CHÚ Ý: Nếu bạn nói Tiếng Việt, có các dịch vụ hỗ trợ ngôn ngữ miễn phí dành cho bạn. Gọi số 1-720-777-9800.

주의: 한국어를 사용하시는 경우, 언어 지원 서비스를 무료로 이용하실 수 있습니다. 1-720-777-9800 번으로 전화해 주십시오

注意：如果您使用繁體中文，您可以免費獲得語言援助服務。請致電1-720-777-9800。

ВНИМАНИЕ: Если вы говорите на русском языке, то вам доступны бесплатные услуги перевода. Звоните 1-720-777-9800.

ማስታወሻ: የሚናገሩት ቋንቋ ኣማርኛ ከሆነ የትርጉም እርዳታ ድርጅቶች፣ በነጻ ሊያግዝዎት ተዘጋጀተዋል፡ ወደ ሚከተለው ቁጥር ይደውሉ 1-720-777-9800 (መስማት ለተሳናቸው.

ملحوظة: إذا كنت تتحدث اذكر اللغة، فإن خدمات المساعدة اللغوية تتوافر لك بالمجان. اتصل برقم 1-720-777-9800 (رقم .

ACHTUNG: Wenn Sie Deutsch sprechen, stehen Ihnen kostenlos sprachliche Hilfsdienstleistungen zur Verfügung. Rufnummer: 1-720-777-9800.

ATTENTION : Si vous parlez français, des services d'aide linguistique vous sont proposés gratuitement. Appelez le 1-720-777-9800.

ध्यान दनु होस:तपाइले नेपाल बोल्नहन्छ भन तपाइको निम्त भाषा सहायता सवाहरू न:शल्क रूपमा उपलब्ध छ । फोन गनु होसर् 1-720-777-9800 ।

PAUNAWA: Kung nagsasalita ka ng Tagalog, maaari kang gumamit ng mga serbisyo ng tulong sa wika nang walang bayad. Tumawag sa 1-720-777-9800.

注意事項：日本語を話される場合、無料の言語支援をご利用いただけます。1-720-777-9800 まで、お電話にてご連絡ください。

Ntị: Ọ bụrụ na asụ Ibo, asụsụ aka ọasụ n'efu, defu, aka. Call 1-720-777-9800.

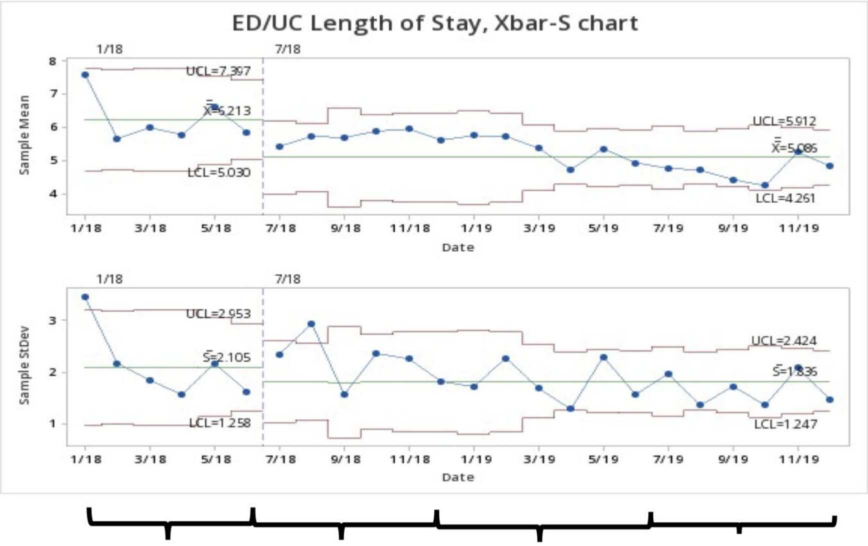

Baseline

Team assembly, stakeholder analysis

Baseline data analysis

Literature review

Kaizen

Pathway development

EMR Tools Development

Pathway Dissemination

Anaphylaxis kits changed
